# Spatiotemporal regulation of MELK during mitosis

**DOI:** 10.3389/fcell.2024.1406940

**Published:** 2024-09-16

**Authors:** Sreemita Majumdar, Song-Tao Liu

**Affiliations:** Department of Biological Sciences, University of Toledo, Toledo, OH, United States

**Keywords:** MELK, KA1 domain, cell cortex, Cdk1, anaphase, PP4

## Abstract

Maternal Embryonic Leucine Zipper Kinase (MELK) has been studied intensively in recent years due to its overexpression in multiple cancers. However, the cell biology of MELK remains less characterized despite its well-documented association with mitosis. Here we report a distinctive pattern of human MELK that translocates from the cytoplasm to cell cortex within 3 min of anaphase onset. The cortex association lasts about 30 min till telophase. The spatiotemporal specific localization of MELK depends on the interaction between its Threonine-Proline (TP) rich domain and kinase associated 1 (KA1) domain, which is regulated by CDK1 kinase and PP4 protein phosphatase. KA1 domains are known to regulate kinase activities through various intramolecular interactions. Our results revealed a new role for KA1 domain to control subcellular localization of a protein kinase.

## 1 Introduction

Maternal Embryonic Leucine Zipper Kinase (MELK) is a member of the AMPK-related protein serine/threonine kinase subfamily, which in turn belongs to the Kin1/PAR-1/MARK family (Gil et al., 1997; Heyer et al., 1997; Tassan and Le Goff, 2004). The kinases in the Kin1/PAR-1/MARK family are conserved from yeast to man and are involved in cell polarity, microtubule dynamics, and cell proliferation (Tassan and Le Goff, 2004). MELK overexpression has been described in many cancers and cancer stem cells (Rhodes et al., 2004; Nakano et al., 2005; Nakano et al., 2008; Nakano and Kornblum, 2009; Pickard et al., 2009; Hebbard et al., 2010; Kappadakunnel et al., 2010; Kuner et al., 2013; Wang et al., 2014; Settleman et al., 2018). These studies suggested upregulated MELK expression is a predictor for poor survival among cancer patients. Indeed, *MELK* is among the clinically used Mammaprint and Prosigna (PAM50) breast cancer signature genes (van de Vijver et al., 2002; Parker et al., 2009; Eroles et al., 2011; Tian et al., 2011). MELK was also ranked #11 in the CIN25 signature genes whose overexpression is characteristic of cancer cells exhibiting chromosomal instability (Carter et al., 2006). Targeting MELK seems a good choice for developing novel cancer therapy. Several MELK small molecule inhibitors have been published, and one of them, OTS167 (formerly OTSSP167), has been tested in multiple Phase I clinical trials (Mahasenan and Li, 2012; Canevari et al., 2013; Chung and Nakamura, 2013; Beke et al., 2015; Toure et al., 2016; Huang et al., 2017; Klaeger et al., 2017; McDonald and Graves, 2020).

However, OTS167 has off-target effects that inhibit multiple kinases involved in the spindle assembly checkpoint signaling and chromosome dynamics (Ji et al., 2016). Furthermore, CRISPR/Cas9 mediated knockout or interference demonstrated that MELK is not essential for proliferation of the mass of cancer cells under many tested conditions (Huang et al., 2017; Lin et al., 2017; Giuliano et al., 2018; Settleman et al., 2018). In addition, off-target effects were also demonstrated for multiple *MELK* shRNAs which had been widely used in previous work (Huang et al., 2017; Lin et al., 2017; Giuliano et al., 2018; Settleman et al., 2018). Controversies concerning MELK functions in cancer development still remain (Janostiak et al., 2017; Jurmeister et al., 2018; Wang et al., 2018; McDonald et al., 2020), but recent advances demand better understanding of MELK functions at the molecular and individual cell levels.

Although it has been indicated in mRNA splicing, apoptosis, DNA damage repair, drug resistance, and many other processes (Vulsteke et al., 2004; Lin et al., 2007; Jung et al., 2008; Choi and Ku, 2011), the protein level, phosphorylation level and kinase activity of endogenous MELK all peak during mitosis (Davezac et al., 2002; Vulsteke et al., 2004; Badouel et al., 2006; Chartrain et al., 2006; Badouel et al., 2010; Le Page et al., 2011; Tipton et al., 2012; Ji et al., 2016). Previously we have found that MELK is co-transcribed with multiple centromere/kinetochore components, which also suggested a role in mitosis regulation (Tipton et al., 2012). Indeed, MELK has been indicated in cytokinesis in several reports (Cordes et al., 2006; Le Page et al., 2011; Wang et al., 2014). However, the cellular level regulation of human MELK during mitosis has not been systematically addressed. We hereby report our results on the unique spatiotemporal localization pattern of MELK during mitosis and its regulation.

## 2 Materials and methods

### 2.1 Cell culture, synchronization, and drug treatment

HeLaM, a subline of HeLa, and HeLaM or MCF7 cell lines stably expressing mRFP-histone H2A were cultured as previously described (Wang et al., 2014). To block cells in G1/S, cells were treated with 2.5 mM thymidine (Sigma-Aldrich) for 24 h. To block cells in prometaphase, cells were treated with 2.5 mM thymidine for 16 h, washed and treated with nocodazole at 0.2 µM (60 ng/mL) for 12 h. OTS167 was a gift from Drs. Yusuke Nakamura, Takuya Tsunoda and Yo Matsuo at Onco Therapy Science and was used at 100 nM (Chung et al., 2012). The proteasome inhibitor MG132 and the CDK1 inhibitor RO-3306 were used at 20 μM and 5 μM final concentrations, respectively. Additional information of these inhibitors and other kinase inhibitors are summarized in Supplementary Table S1.

### 2.2 Immunoblot and immunofluorescence

MELK antibody and immunoblotting procedure were previously described (Ji et al., 2016). To prepare CDK1 inhibitors treated cell lysates used in Figure 4A, HeLa cells were treated with nocodazole at 0.2 µM (60 ng/mL) for 12 h, then RO-3306 or Roscovitine were added to final concentrations of 5 µM together with MG132 (20 µM) for 1 h. Mitotic cells were harvested by shake-off. For immunofluorescence, HeLaM cells were seeded on coverslips, treated with 2.5 mM thymidine for 24 h, washed then directly released into drug-free medium. After 9–10 h when cells were observed to enter mitosis, coverslips were fixed with ice-cold methanol for 20 min at −20°C. The fixed cells were gently washed three times in PBS, blocked in PBS containing 5% BSA for 1 h, and left in MELK primary antibody diluted in the blocking buffer overnight. The AlexaFluor 488 conjugated secondary antibody was used at 1:1,000 and incubated for 30 min. The coverslip was mounted using Fluoroshield containing DAPI (Sigma). The images were collected on a Leica TCS SP8 confocal microscope with a ×63 objective (numerical aperture = 1.40).

### 2.3 Plasmids and transfections

Human MELK cDNA was cloned into pENTR-TOPO vector (Invitrogen) and point mutations were made following the QuikChange site-directed mutagenesis protocol (Agilent). The primers are listed in Supplementary Table S2. The mutants include D150A, T167A, T167E, ^555^RRLK^558^ to SSSS, ^627^RRQR^630^ to SSSS, T446E, and the 5A or 5E mutants (T460, T466, T478, S498, T518 to A or E). All mutants were verified by Sanger sequencing (Genewiz). The wild type and mutants are recombined into an eGFP vector using Gateway LR clonase (Invitrogen). The mCherry-Lifeact-7 was a gift from Michael Davidson (Addgene plasmid # 54491). DNA transfection was carried out using polyethylenimine as described (Ji et al., 2016).

### 2.4 Live cell imaging and quantitation

For live cell imaging, cells were plated on 35 mm dish with a coverslip glued bottom (Cellvis) at about 30% confluence and transfected with the desired constructs the next day. Images were captured ∼24 h later on a Leica SP8 confocal microscope usually with 3 min intervals in 2 μm Z stacks spanning the cell dimension. The imaging DMEM medium contains 20 mM HEPES (pH 7.4) but no phenol red, and cells were maintained in an on-stage heating chamber set at 37°C. To quantify cell cortex localized GFP-MELK, the cells were stained with CellBrite Steady 650 Membrane Staining dye (Biotium) to delimit cell membrane. The middle plane of image stacks was selected for quantification. Images were analyzed using the membrane stain to create a mask and apply to the GFP channel. To measure GFP intensity at the cortex in experiments without membrane dye staining as shown in Figures 4, 5, a 1 µm width line was drawn around the cell edge in ImageJ (Schneider et al., 2012). After subtracting background, GFP intensity measured along the line was considered as cortex-localized signals, and the internal circle was measured as cytoplasm. The % of GFP intensity at the cortex was compared to total GFP intensity (cortex + cytoplasm) in the cell.

### 2.5 Statistical analysis

Statistical analysis was performed using GraphPad Prism software (version 10.1.0). Data is presented as mean ± SD. The student’s t-test was used to assess the significance of differences between two samples. For multiple samples, 2-way ANOVA with Tukey’s multi comparison test was used (for Figures 4, 5 experiments).

## 3 Results

### 3.1 MELK is translocated to cell cortex upon the metaphase-to-anaphase transition

**FIGURE 1:**
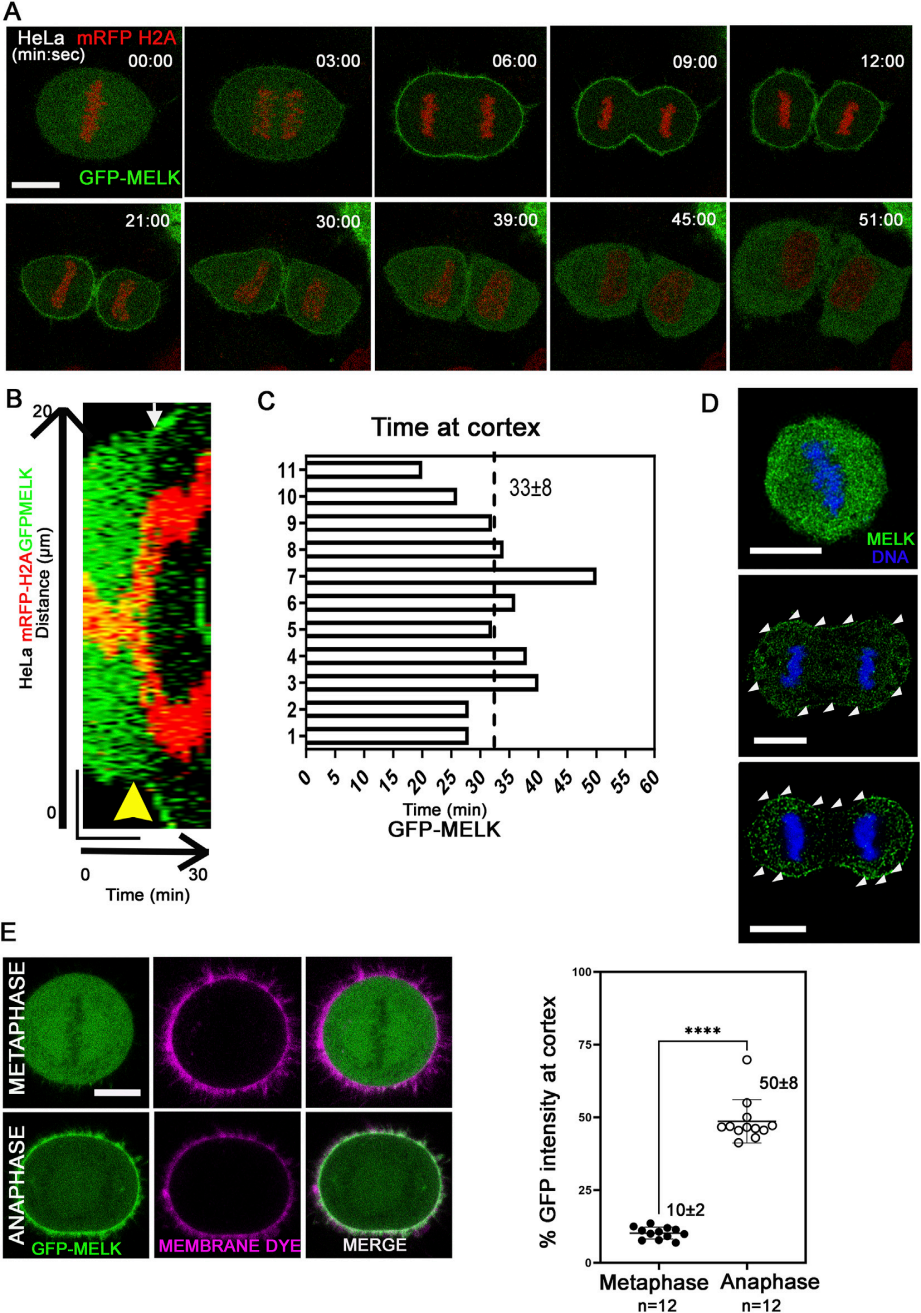
MELK is translocated to cell cortex upon the metaphase-to-anaphase transition **(A)** Selected images from a time lapse recording from metaphase to cytokinesis of a GFP-MELK transfected HeLa cell that stably expresses mRFP-histone H2A. Single plane images are shown with time stamps marking min:sec. The last metaphase image is considered as time 0. Scale bar is 10 µm. **(B)** The kymograph shows GFP-MELK and mRFP-histone H2A signals from a representative movie for 30 min, with a bar of 20 µm drawn across the cell. The yellow arrowhead points to anaphase onset and the white arrow points to the beginning of GFP cortical translocation. **(C)** Bar graphs show the durations of GFP-MELK residing at the cell cortex. The last metaphase image is considered as time 0. **(D)** Immunofluorescence of MELK (green) in metaphase (top) and anaphase cells (middle and bottom) with DNA counterstained with DAPI (blue). Arrowheads (white) point to MELK signals along the cell cortex. Bar = 10 µm. **(E)** The intensities of GFP signals in the whole cell or at cell cortex are measured in metaphase and anaphase cells, with the cortex delimited by a membrane lipid staining fluorescent dye (CellBrite Steady 650). Representative images are shown on the left, and the quantitation shown as a scatter plot on the right. **** denotes P < 0.0001. Scale bar is 10 µm.

Previously it was found that MELK re-localizes to the cell cortex in anaphase and telophase *Xenopus* or HeLa cells (Chartrain et al., 2006; Le Page et al., 2011; Tipton et al., 2012). To gain more insights into the translocation, live cell imaging was used to track GFP-MELK localization in a HeLa cell line stably expressing mRFP-tagged histone H2A (Figures 1A–C). GFP-MELK primarily resides in the cytoplasm until metaphase but translocates to the cell cortex within ∼3 min after the metaphase-to-anaphase transition and remains associated until late telophase. The cortex localization for MELK lasts 33 ± 8 min (mean ± SD, n = 11) during anaphase and telophase (Figure 1C). Endogenous MELK was also found to localize at cell cortex in anaphase cells by immunofluorescence (Figure 1D), similarly as reported before (Chartrain et al., 2006). Translocation of MELK to cell cortex during anaphase cells has also been observed in other cell lines including MCF-7 and hTERT-RPE1, suggesting it is a common feature for MELK regulation (Supplementary Figure S1). We checked the GFP-MELK localization in interphase especially in early G1 and late G2 phases and found GFP signals were enriched in the cytoplasm with no clear cell cortex localization (Supplementary Figure S2A).

Using a lipid binding fluorescent dye to delimit the cell membrane, the GFP intensity of MELK at the cortex was quantified (Figure 1E). The GFP intensity localized at the cell cortex increased from 10% ± 2% (n = 12 cells) of the total GFP intensity in metaphase to 50% ± 8% (n = 12 cells) in anaphase. Line scans drawn across the cortex in metaphase and anaphase cells stained with the lipid binding dye supported MELK localization to the membrane during anaphase (Supplementary Figure S2B). Actin cytoskeleton is a major component of cell cortex (Kunda and Baum, 2009). Live cell fluorescence microscopy was performed in cells transfected with Lifeact that binds to filamentous actin (F-actin) (Riedl et al., 2008; Belyy et al., 2020). The temporal control of MELK was indicated by GFP-MELK co-localization with LifeAct signals in anaphase but not metaphase cells (Supplementary Figure S3). This is consistent with the earlier result that cortical MELK co-localized with filamentous actin in fixed samples (Chartrain et al., 2006). The above results supported that MELK translocates from the cytoplasm to cell cortex within ∼3 min of anaphase onset.

### 3.2 Reduced CDK1 activity is required for MELK cortex localization

**FIGURE 2:**
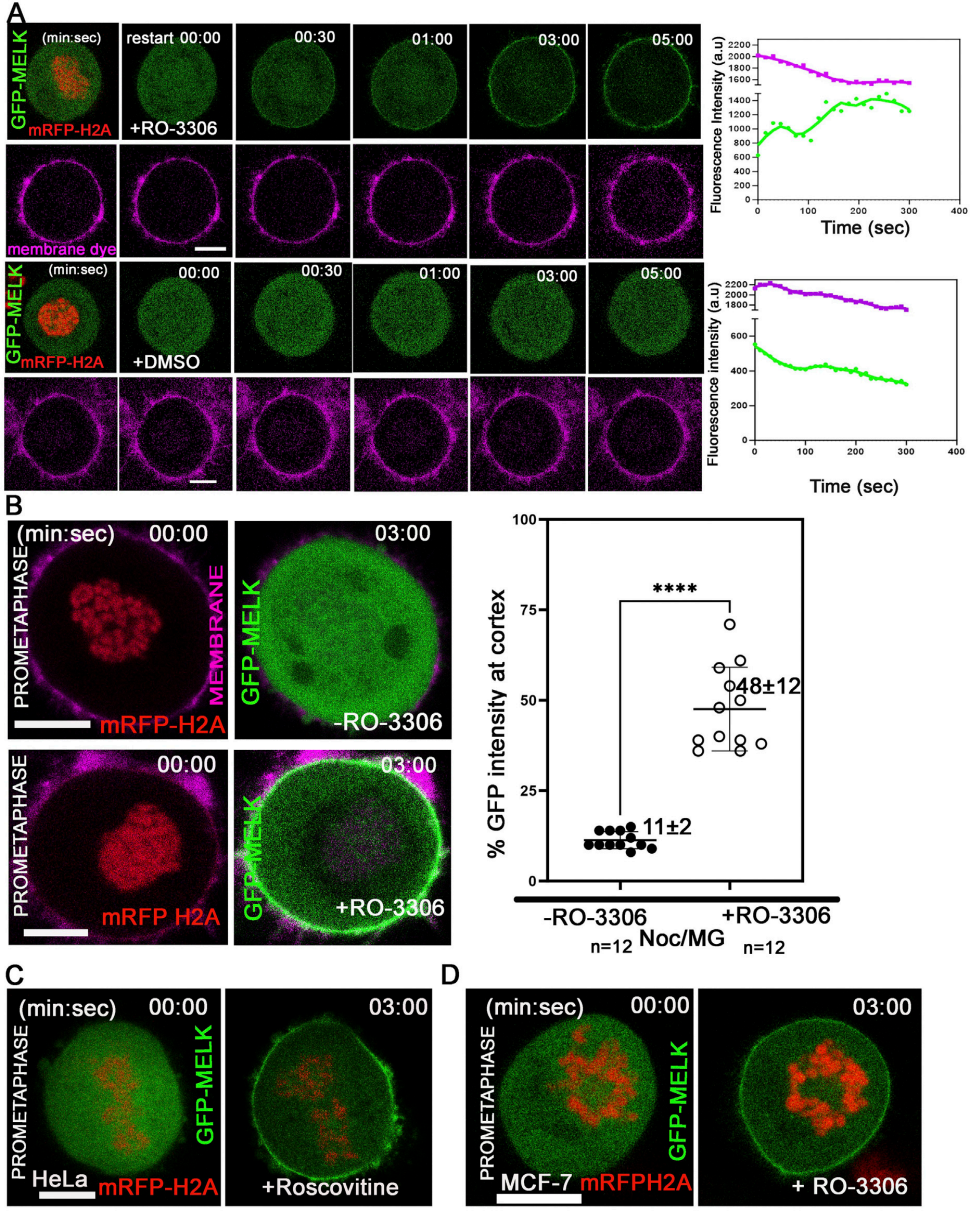
Reduced CDK1 activity is required for MELK cortex localization. **(A)** HeLa cells expressing mRFP-histone H2A were arrested in prometaphase with nocodazole and MG132 and stained with membrane dye (CellBrite Steady 650) before imaging. RO-3306 was added and live cell imaging immediately started with a time interval of 10 s. GFP-MELK and mRFP-H2A (1st row) and the membrane dye (2nd row) are shown in green, red and magenta, respectively. The intensities of cortical GFP and membrane dye were quantified with respect to time. The time stamps indicate minutes: seconds (min: sec). The bottom two rows are mock experiments with DMSO added. **(B)** The intensities of GFP signals in the whole cell or at cell cortex were measured before or 3 min after addition of RO-3306 in HeLa cells expressing mRFP-histone H2A, transfected with GFP-MELK and arrested in prometaphase by nocodazole and MG132 treatment (Noc/MG). Representative images are shown on the left, and the quantitation shown as a scatter plot on the right. Scale bar is 10 µm. **** indicated P < 0.0001 in Student’s t-test. **(C)** HeLa cells transfected with GFP-MELK were treated similarly as in **(A)** but exposed to Roscovitine (5 µM) for CDK1 inhibition**.** A single plane representative image is shown before and after treatment. **(D)** MCF-7 cells expressing mRFP-histone H2A were treated similarly as in **(A)** with RO-3306. A single plane representative image is shown before and after treatment. Scale bar = 10 µm.

The temporally restricted cortex localization of MELK during late mitosis suggested that MELK localization is under control of mitotic kinases. Since the translocation occurs after the anaphase onset, it is hypothesized that the drop in CDK1 kinase activity regulates MELK translocation. To test the hypothesis, HeLa cells stably expressing mRFP-histone H2A were transfected with GFP-MELK, and treated with nocodazole and MG132 to arrest cells in prometaphase. When exposed to CDK1 inhibitor RO-3306 (Vassilev et al., 2006), GFP-MELK localized to the cortex within 2–3 min and became stable at the cortex onwards, even though the cells stayed in prometaphase-like state based on chromosome configuration (Figures 2A,B). Quantitation found that 48% ± 12% of GFP signals (n = 12 cells) got enriched at the cell membrane after 3 min of RO-3306 treatment (Figure 2B). Similar results were observed using another CDK1 inhibitor roscovitine (Meijer et al., 1997) or using MCF-7- mRFP H2A cell line (Figures 2C,D). This showed that reduced CDK1 activity triggers MELK localization onto the cortex.

We have shown before that inhibiting mitotic kinases Plk1, Aurora B or MPS1 did not prematurely target MELK to the cortex (Ji et al., 2016). We expanded the test and found inhibiting Src/Abl kinases (PD166326), Aurora A kinase (MLN8237), p38 MAPK (SB202190), MEK1 (PD98059), JNK (JNK-IN-8), and MEK1/2 (U0126) also did not affect the timing of cortex association of GFP-MELK (Supplementary Figure S4).

We confirmed that OTS167 exposure also prematurely targeted MELK to the cortex (Ji et al., 2016) (Supplementary Figure S4, third row, part I). Although OTS167 was suggested to be a MELK specific inhibitor, later results found it promiscuously inhibits many other kinases (Ji et al., 2016; Huang et al., 2017; Klaeger et al., 2017; Giuliano et al., 2018). Applying two more specific MELK inhibitors, HTH-01-091 and NVS-MELK8a, revealed no premature localization of GFP-MELK in the same assay (Supplementary Figure S4, bottom row) (Toure et al., 2016; Huang et al., 2017). In addition, two kinase dead mutants of MELK (D150A or N137A), when fused with GFP, displayed similar translocation kinetics as wild type MELK (Supplementary Figure S5). Other MELK kinase mutants such as T167A and T167E mutants at the key T167 residue in the activation loop (Cao et al., 2013) did not alter the localization pattern either (Supplementary Figure S5). These results suggested that MELK kinase activity is likely not required for its own localization pattern.

### 3.3 The KA1 domain is required for MELK cortex localization

To further understand the cortex localization of MELK, we next shifted attention to its kinase associated 1 (KA1) domain. KA1 domains exist primarily in kinases in the Kin1/PAR-1/MARK family, but also in other kinases such as Chk1 and RNA processing enzymes (Moravcevic et al., 2010; Gong et al., 2018; Paung and Seeliger, 2018; Aoyama et al., 2020; Ju et al., 2023). The KA1 domains in the MARK family show plasma membrane localization in *S. cerevisiae*, *S. pombe* and mammalian cells, and MARK1-KA1 binds to acidic phospholipids in cells and *in vitro* (Moravcevic et al., 2010; Rincon et al., 2014; Emptage et al., 2017a).

**FIGURE 3:**
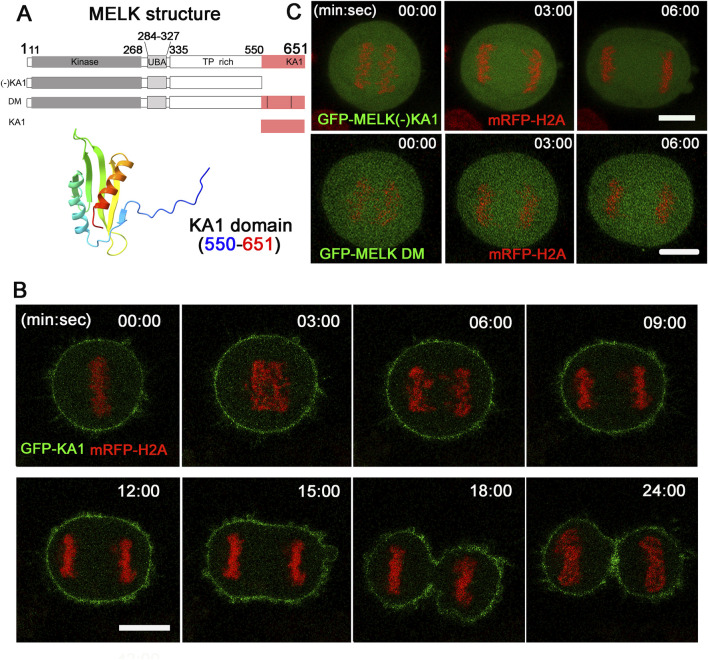
The KA1 domain is required for MELK cortex localization. **(A)** Shown on the top are diagrammatic representations of full-length MELK with its kinase, UBA, TP rich and KA1 domains, MELK truncation with no KA1 domain [(−)KA1], MELK double mutant (DM) in the KA1 domain (^555^RRLK^558^ to SSSS, and ^627^RRQR^630^ to SSSS), and only KA1 domain. The numbers indicate the residues delimiting each domain. AlphaFold predicted structure of the MELK-KA1 domain (550–651) is shown below. **(B)** Images from time-lapse microscopy of HeLa mRFP-histone H2A cells transfected with GFP-KA1. Time stamp, min: sec with last metaphase plate set as t = 0. Scale bar = 10 µm. **(C)** HeLa cells expressing mRFP-histone H2A were transfected with GFP-MELK (−) KA1 truncation (upper panel) and GFP-MELK double mutant (DM, lower panel). Representative images in anaphase are shown. Scale bar is 10 µm.

Alphafold predicted that human MELK KA1 has similar fold as other solved KA1 structures (Meng et al., 2023) (Figure 3A). The MELK-KA1 domain (550–651 residues) has a theoretical isoelectric point: *pI* = 9.330 and at physiological pH 7.4: *z* = +5.886. GFP-MELK-KA1 is indeed localized to the cortex throughout the cell cycle (Supplementary Figure S6), in contrast to full length MELK. Live cell imaging of GFP-KA1 domain confirmed persistent localization at cortex form metaphase to the end of cytokinesis (Figure 3B).

To further investigate the requirement of KA1 domain for MELK association with the cell cortex, a KA1-truncated MELK was fused with GFP and transfected into HeLa cells. As shown in Figure 3C, no translocation was observed even during anaphase. Two conserved basic patches in the KA1 domains of the MARK family kinases were found to be essential for membrane association through directly binding to phospholipids (Moravcevic et al., 2010; Emptage et al., 2017a; Emptage et al., 2017b). When corresponding patches in human MELK (^555^RRLK^558^ and ^627^RRQR^630^) were mutated, the resulted GFP-MELK-KA1 double mutant (“DM”) also failed to re-locate to the cortex in anaphase cells (Figure 3C). The same DNA constructs showed the same localization patterns despite differential expression levels in individual cells (Supplementary Figure S7). These results supported the idea that the KA1 domain particularly its two conserved basic patches provide the physical foundation of MELK association with the cell cortex.

### 3.4 The phosphorylation status of the TP region regulates KA1 and localization of MELK

**FIGURE 4:**
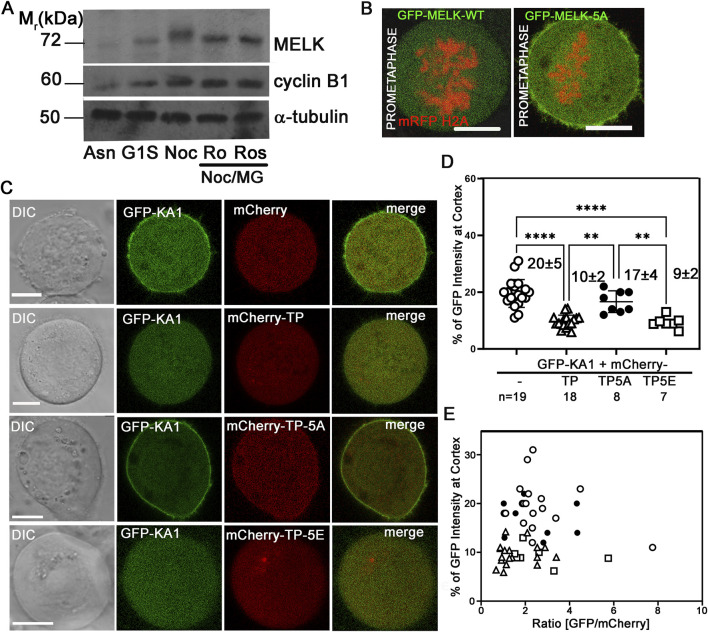
Phosphorylation status of TP domain affects MELK cortex localization. **(A)** Western Blot analysis of MELK in HeLa cell lysates after 1 h treatment with RO-3306 (Ro) and Roscovitine (Ros) on Nocodazole/MG132 (Noc/MG) arrested cells. Also shown are lysates from asynchronized cells (Asn), thymidine (G1S) or nocodazole (Noc) arrested cells. Cyclin B1 and α-tubulin were also probed to indicate mitotic stage and as a loading control, respectively. **(B)** GFP fused with wild type (WT) MELK or GFP-MELK-5A (mutant of 5 S/T to A on presumable CDK1 sites) were transfected in HeLa cells expressing mRFP-histone H2A and arrested at prometaphase by treatment with nocodazole and MG132. Single plane still images are shown. Scale bar is 10 µm. **(C)** HeLa cells were co-transfected with GFP-KA1 domain and mCherry or mCherry-fused different forms of TP domain (mCherry-TP, mCherry-TP-5A, or mCherry-TP-5E), and arrested in prometaphase. Representative single plane still images are shown. Scale bar = 10 µm. **(D)** Quantification of cortex localized GFP-KA1 signals when different mCherry constructs were co-transfected as in **(C)**. ** denotes P < 0.01 and **** indicated P < 0.0001 **(E)** Quantification of cortex localized GFP-KA1 signals versus relative GFP/mCherry intensity ratios for all cells quantified in **(D)**.

We then hypothesized that CDK1 activity temporally controls MELK localization through phosphorylating MELK and preventing its KA1 binding to cell membrane during prometaphase. Human MELK contains a so-called Threonine-Proline (TP) rich unstructured region (335–550 amino acids) between its kinase-UBA domain and KA1 domain, whose TP rich composition is not shared with other MARK family kinases (Vulsteke et al., 2004) (Figure 3A). The MELK-TP region contains 10 TP and 1 SP sites with five of them conforming the CDK1 substrate consensus motif S/T-P-x-K/R (Songyang et al., 1994). Phosphoproteomics studies have confirmed *in vivo* phosphorylation of several sites in the TP region and several sites were proposed to be directly phosphorylated by CDK1 (Badouel et al., 2006; Hornbeck et al., 2015). Western blot of cell lysates prepared from nocodazole and MG132 arrested HeLa cells showed reduced MELK mobility shift after treatment with RO-3306 or Roscovitine, two CDK1 inhibitors, supporting CDK1 phosphorylation of MELK (Figure 4A). Although some earlier reports indicated that RO-3306 could reduce CDK1 after long time treatment, under our experimental conditions, RO-3306 did not affect CDK1 level but inhibit its kinase activity (Supplementary Figure S8). The serine/threonine residues in the five conserved (S/T)P sites within the TP region (^460^TPNR^463^, ^466^TPSK^469^, ^478^TPIK^481^, ^498^SPER^501^ and ^518^TPKR^521^) were mutated to alanines to create a phosphoresistant 5A mutant in otherwise full length MELK. Six out of 7 cells transfected with GFP-MELK-5A prematurely localized GFP to cell cortex in prometaphase cells, in contrast to GFP-MELK wild type transfected cells (Figure 4B), indicating the importance of phosphorylation status of the five (S/T)P sites.

To further probe the effect of TP phosphorylation, we co-transfected HeLa cells with GFP-KA1 domain and mCherry fused wild type TP fragment or TP-5A (phosphoresistant) or TP-5E (phosphomimetic) mutants and arrested cells in prometaphase by treatment with nocodazole and MG132. As shown in representative images in Figure 4C, ∼20% of GFP-KA1 is localized to cell cortex when co-transfected with mCherry vector in prometaphase cells. The cortex fraction of GFP-KA1 drops to 10% when mCherry-TP was co-expressed. Interestingly when mCherry-TP-5A was expressed, accumulation of GFP-KA1 at the cell cortex was observed again. Conversely, when mCherry-TP-5E was expressed, GFP-KA1 localization to the cortex was comparable as in mCherry-TP co-expressed cells (Figures 4D, E). Despite variations of mCherry and GFP expression levels, only TP-5A co-expressed cells but not TP or TP-5E expressed cells showed detectable cell cortex accumulation of GFP-KA1 signals. The series of experiments are consistent with the idea that phosphorylation in the MELK TP region by CDK1 could affect the interactions between TP and the KA1 domain, hence affecting KA1 availability for phospholipid binding which further controls the timing of MELK cortex localization.

### 3.5 PP4 protein phosphatase is likely to regulate MELK cortex localization

**FIGURE 5:**
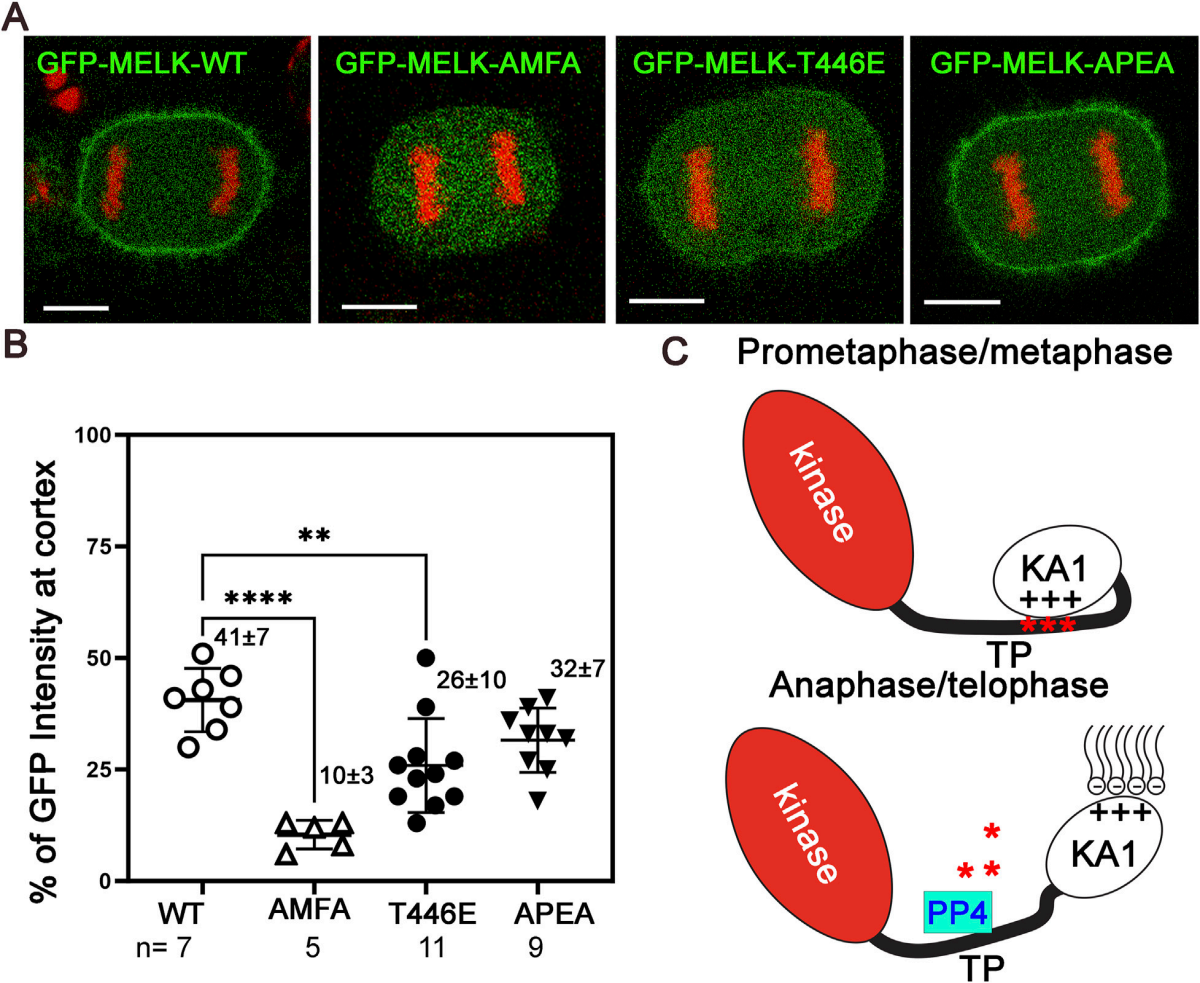
PP4 helps determine the timing of MELK cortex localization. **(A)** Selected images of anaphase HeLa cells transfected with GFP-MELK-wild type (WT), or its FPEP to APEA mutant, or FMFP to AMFA mutant, or T446E mutant to show differences in the cortex localization of MELK. These cells also stably express mRFP-histone H2A (shown in red). **(B)** Scatter plot showing the % GFP fluorescence intensity at the cortex in anaphase cells transfected with different GFP-MELK constructs. The last anaphase images with no detectable cleavage furrow signs were used for quantification. ** denotes P < 0.01 and **** indicated P < 0.0001. **(C)** A model to explain the spatiotemporal control of MELK during mitosis. See text for details.

Many mitotic phosphoproteins are dephosphorylated by protein phosphatases as cells exit from mitosis (Holder et al., 2019; Nilsson, 2019). Recently Ueki et al. identified two overlapping potential binding motifs (FXXP) on MELK for PP4 protein phosphatase: FMFP and FPEP within ^439^FMFPEP^444^ in the MELK TP region (Ueki et al., 2019) (Supplementary Figure S9A). We wonder whether PP4 dephosphorylates MELK TP region and triggers MELK cortex localization. To test the idea, we mutated FMFP and FPEP to AMFA and APEA respectively in GFP-MELK. When transfected into HeLa-mRFP-H2A cells, the APEA mutant showed 32% ± 8% (n = 9) cortex localized GFP, similarly to 41% ± 7% (n = 7) in wild type MELK transfected anaphase cells (Figure 5; Supplementary Figure S10). However, the AMFA mutant only had 10% ± 3% (n = 5) GFP translocated to the anaphase cortex (Figure 5; Supplementary Figure S10). This indicated that the FMFP motif is required for MELK anaphase cortex translocation, probably through recruiting PP4. PP4 binding to the FXXP motif can be negatively impacted by adjacent phosphorylation (Ueki et al., 2019). T446 is an *in vivo* phosphorylation site adjacent to the FMFP motif (Hornbeck et al., 2015). We therefore tested the T446E mutant, and found the phosphomimetic mutant also reduced anaphase cortex GFP signals, although to a lesser degree, to 26 ± 10 (n = 11) (Figure 5; Supplementary Figure S10). We tested but did not find significant PP4 catalytic subunit in the MELK immunoprecipitates using either prometaphase or anaphase cell lysates (Supplementary Figure S9B). These results indicate that PP4 is a strong candidate phosphatase to counter CDK1 phosphorylation of MELK TP region, hence contributing to timing the MELK anaphase cortex translocation. However, the PP4-MELK interaction might be transient.

## 4 Discussion

Recent years have witnessed interest and confusion in MELK as a potential target for cancer therapy due to its overexpression in multiple cancers (Ganguly et al., 2014; Settleman et al., 2018; McDonald and Graves, 2020). However fundamental biology of MELK at cellular level has lagged behind despite its protein level, phosphorylation and kinase activity was known to peak during mitosis. In this work we provided an updated quantitative view about the cell cortex translocation of MELK shortly after anaphase onset, and presented molecular explanations underlying this unique spatiotemporal localization pattern during mitosis. Functional studies of MELK activities during mitosis are currently ongoing and will be reported in the future.

We propose a model to explain the human MELK localization pattern during mitosis (Figure 5C). In addition to the kinase domain and the UBA domain that helps maintain kinase activity, MELK also contains a KA1 domain, and a disordered TP rich region that is distinct from other MARK family kinases. The KA1 domain was known to bind to acidic phospholipids (Moravcevic et al., 2010; Emptage et al., 2017a). During prometaphase and metaphase, the unstructured TP rich domain of MELK is phosphorylated due to high CDK1 activity. Phosphorylated TP domain especially the portion containing the five (S/T)P sites between 460–521 residues could use clustered negative charges to compete with phospholipids, bind with MELK-KA1 domain, and hence retain MELK in the cytoplasm (Figure 4). The intramolecular TP-KA1 interaction is probably mediated through the two stretches of positively charged basic residues in KA1 domain, which are also essential for binding to phospholipids (Emptage et al., 2017b). Upon anaphase onset, the CDK1 kinase activity is reduced while phosphatases become more active (Holder et al., 2019; Nilsson, 2019). We provided evidence that PP4 contributes to dephosphorylation of MELK (Figure 5; Supplementary Figure S10). Dephosphorylated TP domain cannot effectively interact with KA1, thus releasing the KA1 domain to interact with phospholipids and translocating MELK to the cell cortex. Our imaging results supported the roles of phosphorylation at T460, T466, T478, S498 and T518 in regulating interactions with KA1 domain hence the timing of MELK cortex localization, but we cannot exclude contributions from additional sites such those other 6 TP sites in the TP domain to this process.

The model suggests a new role for KA1 domain to control cell cycle specific subcellular localization of a protein kinase. The model also raises additional questions for future studies. For example, why does full length MELK not go to the cell cortex in interphase cells which should also exhibit lower CDK activity? The relatively lower protein level of MELK during interphase cells might affect its localization, but other mechanisms cannot be excluded. For example, MARK3 cytoplasmic localization was known to be controlled by its interactions with 14-3-3 proteins (Goransson et al., 2006). In addition, cell-cell junctions might also regulate interphase MELK localization as reported for *Xenopus* MELK (Chartrain et al., 2013). Along the same line, GFP-KA1 has distinctive nuclear localization in interphase cells most likely due to the similarity of its two stretches of basic residues to classical nuclear localization signal (Supplementary Figure S6) (Chartrain et al., 2006). However, full length MELK is primarily cytoplasmic in interphase cells (Supplementary Figure S2A), also indicating additional regulation. One possible regulatory mechanism might be interaction between the KA1 and kinase domains, similarly as observed in MARK1 as a way to exert autoinhibition (Emptage et al., 2017a; Emptage et al., 2018). The KA1 mediated autoinhibition of MARK1 also requires the basic patches. If it remains true for MELK, the KA1-kinase domain interaction could cause mutual masking, explaining both low kinase activity and lack of cell cortex localization of MELK during interphase. How MELK KA1 domain switches from intramolecular interaction partners (kinase domain or phospho-TP region) to phospholipids during different cell cycle stages need more clarification in the future. Similarly, the functional consequences of the spatiotemporal specific MELK localization on MELK kinase activity or substrate access are also future research subjects.

## Data Availability

The original contributions presented in the study are included in the article/Supplementary Material, further inquiries can be directed to the corresponding author.
