# Supplementary material for: Spatiotemporal regulation of MELK during mitosis
Source: Front Cell Dev Biol. 2024 Sep 16;12:1406940. doi: 10.3389/fcell.2024.1406940 (PMC11443572; doi:10.3389/fcell.2024.1406940)
Supplement: Supplementary file 1 [file Table1.docx]

**Supplemental Figures**

S1. GFP-MELK is translocated to cell cortex during anaphase in MCF-7 and RPE1 cells.

S2. Additional data on MELK localization in different phases of cell cycle.

S3. Co-localization of GFP-MELK and Lifeact in anaphase cells.

S4. Effects of various kinase inhibitors on the localization of GFP-MELK in cells arrested in prometaphase.

S5. Cortical localization of various mutants of MELK.

S6. MELK-KA1 domain is associated with the cell cortex throughout the cell cycle.

S7. GFP quantification for Fig 3 experiments.

S8. Related to Fig 4. RO-3306 treatment for 1 hr of mitotic cells arrested in nocodazole/MG132 (Noc/MG) did not change CDK1 protein level.

S9. Related to Fig 5. Examination of potential interactions between PP4 and MELK.

S10. Related to Fig 5. Selected images of MELK mutants at potential PP4 binding motifs or a regulatory site.

**Supplemental Tables**

Table S1. List of small molecule inhibitors

Table S2. List of primers


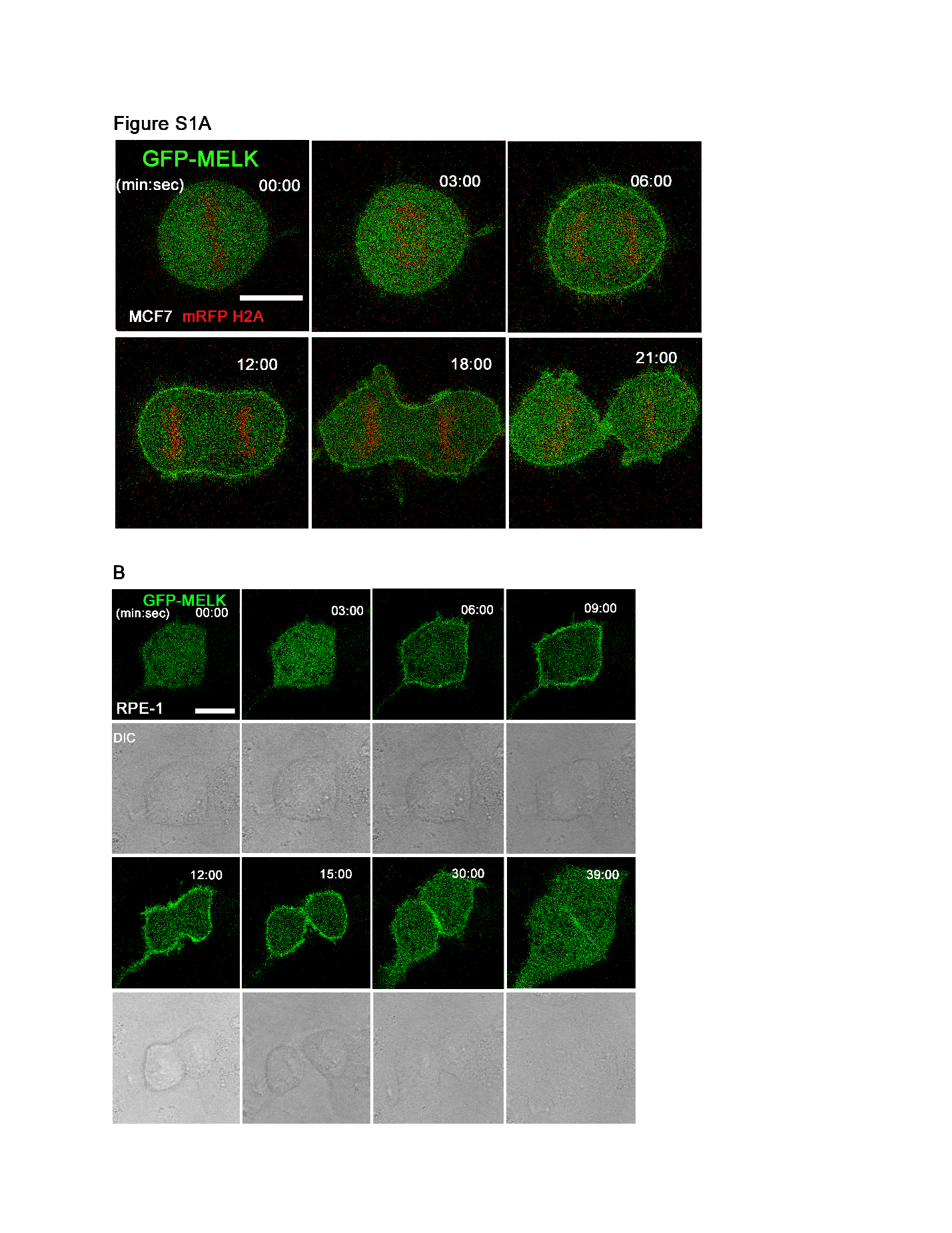


**Figure S1. MELK localization at cortex in anaphase MCF-7 and RPE1 cells.**

Images from time-lapse recordings of a MCF-7 cell stably expressing mRFP-H2A (A) or an hTERT-RPE1 cell (B) transfected with GFP-MELK. Single plane images are shown with time stamp marking min: sec, where the last metaphase image is considered as time 0. Scale bar is 10µm.


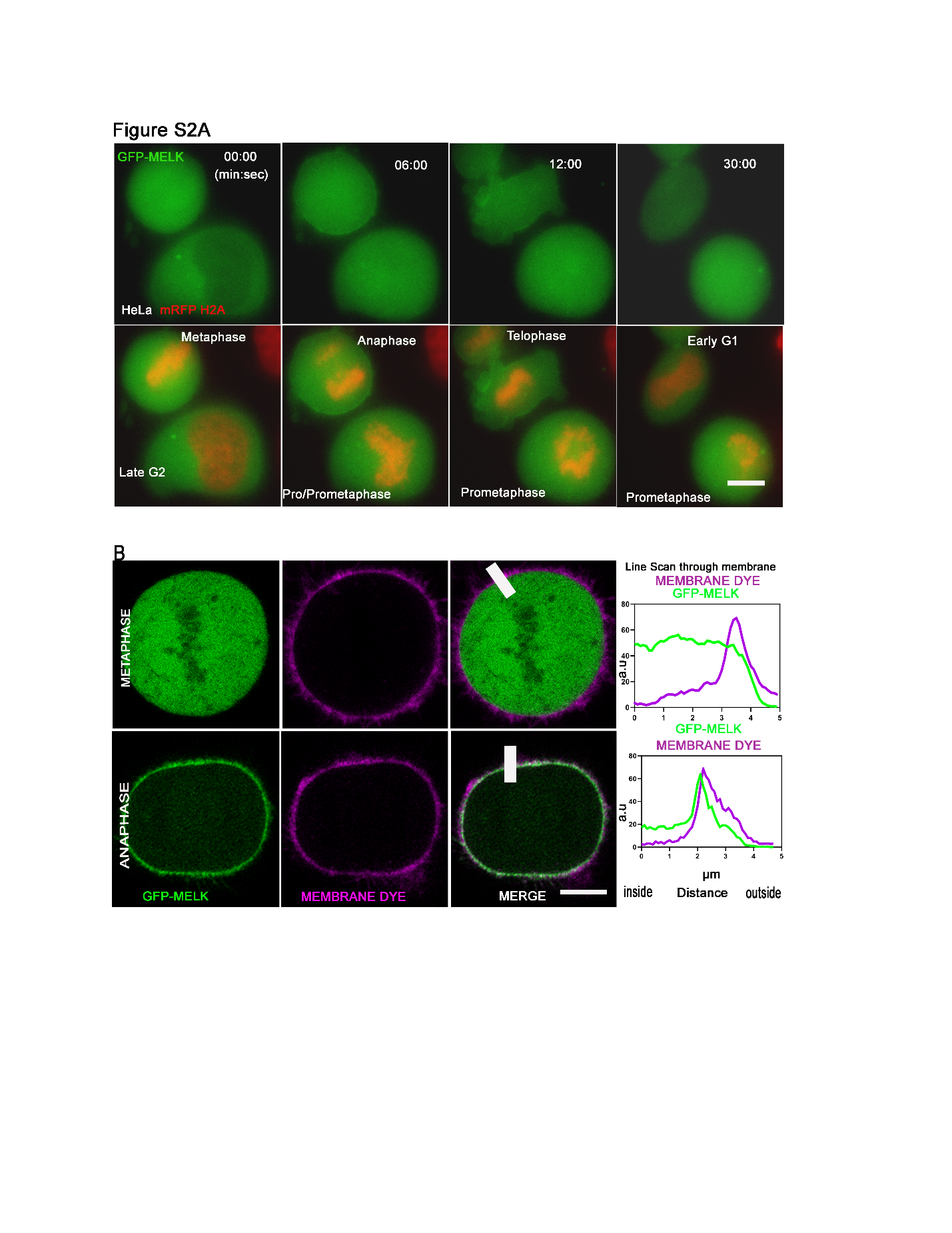


**Figure S2. Additional data on MELK localization in different phases of cell cycle.**

(A) Images from time-lapse recordings of two GFP-MELK transfected HeLa cells stably expressing mRFP H2A, with one showing the G2 transition into mitosis (bottom, time points 00:00 and 06:00), and the other showing transitioning from mitosis to early G1 (top, time points 12:00 and 30:00).

(B) Line scans showing GFP-MELK (green) and membrane dye (magenta) signals across the cell membrane in metaphase (top) and anaphase (bottom) cells.

**
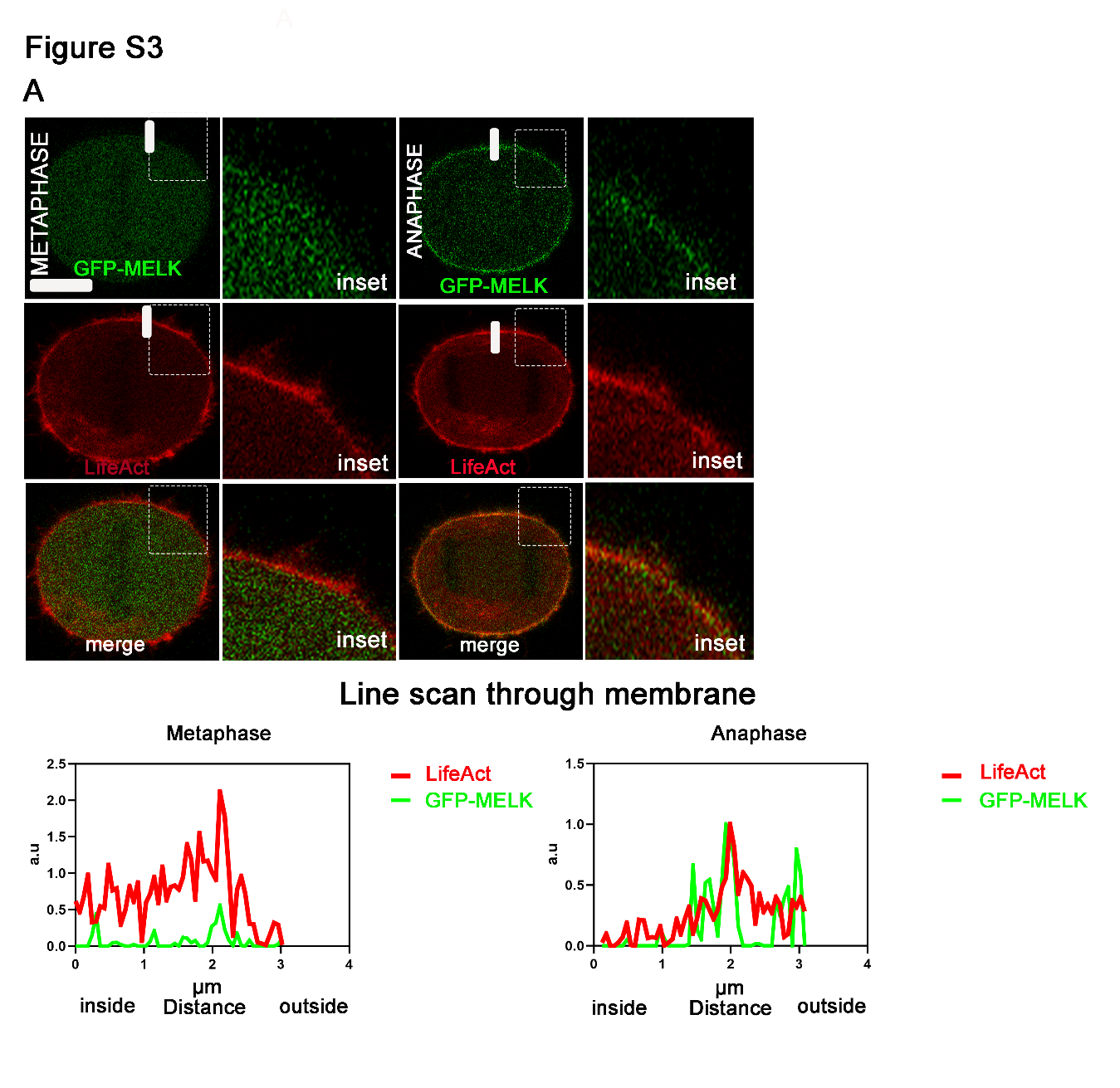
**

**Figure S3. Co-localization of GFP-MELK and Lifeact in anaphase cells.**

Images of a metaphase and an anaphase Hela cell transfected with GFP-MELK and mCherry-Lifeact are shown, with insets for details and line scans indicating co-localization in anaphase cells.

**
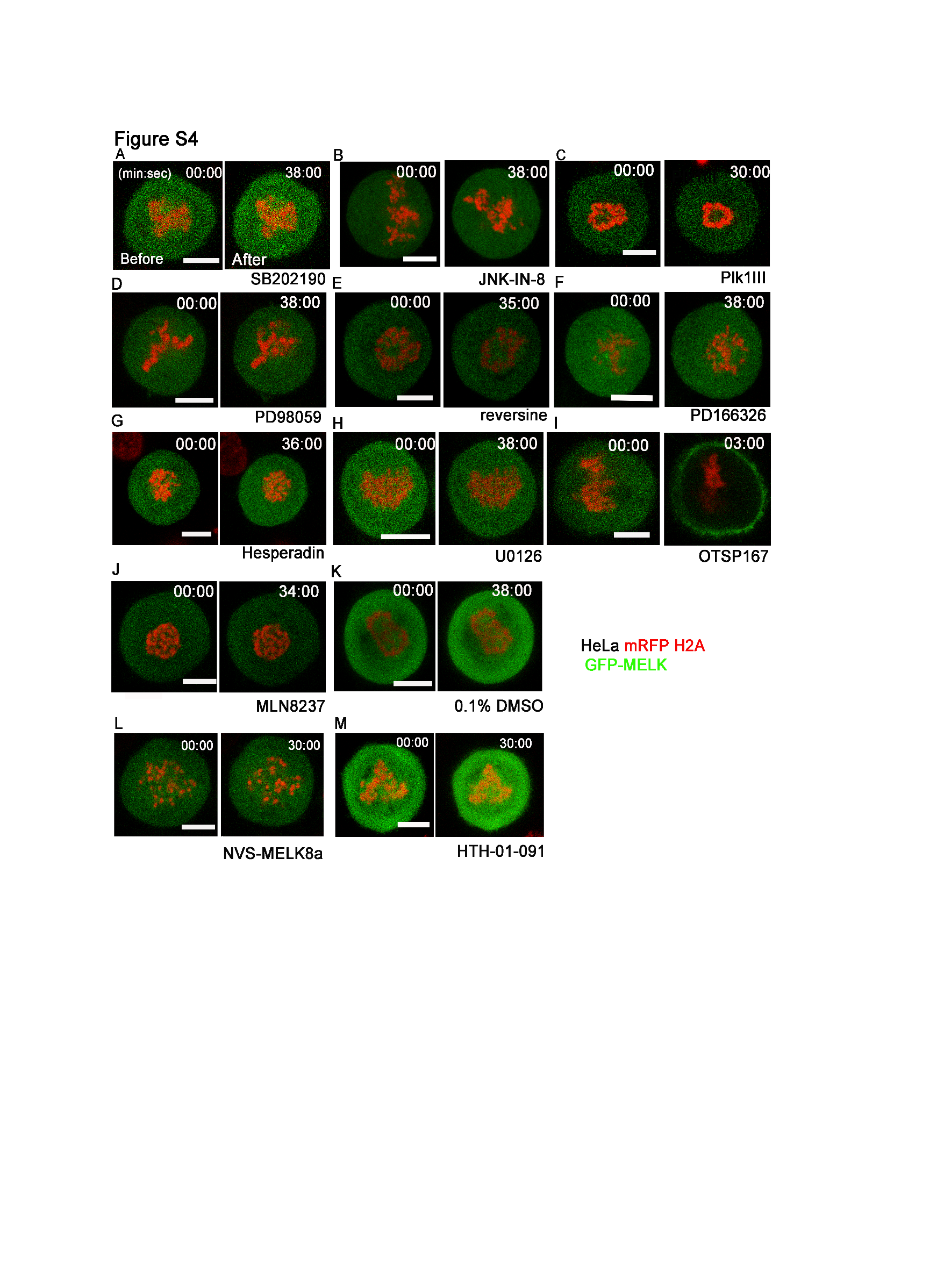
**

**Figure S4. Effects of various kinase inhibitors on the localization of GFP-MELK in cells.** Related to Figure 2. HeLa cells expressing mRFP-H2A and transfected with GFP-MELK were arrested in prometaphase with nocodazole and MG132. These cells were imaged after exposing to different kinase inhibitors. The inhibitors tested are SB202190 (p38 MAPK kinase), JNK-IN-8 (JNK kinases), Plk1 inhibitor III (Plk1 kinase), PD98059 (MEK1 kinase), reversine (MPS1 kinase), PD166326 (Src/Abl kinases), Hesperadin (Aurora B kinase), U0126 (MEK kinases), OTSP167 (MELK and other kinases), MLN8237 (Aurora A kinase), DMSO control, NVS-MELK8a and HTH-01-091 (MELK inhibitors). Single-plane images are shown before (t=0) and after (t~40min) addition of respective inhibitors. Time stamp, min: sec. Scale bar is 10µm.


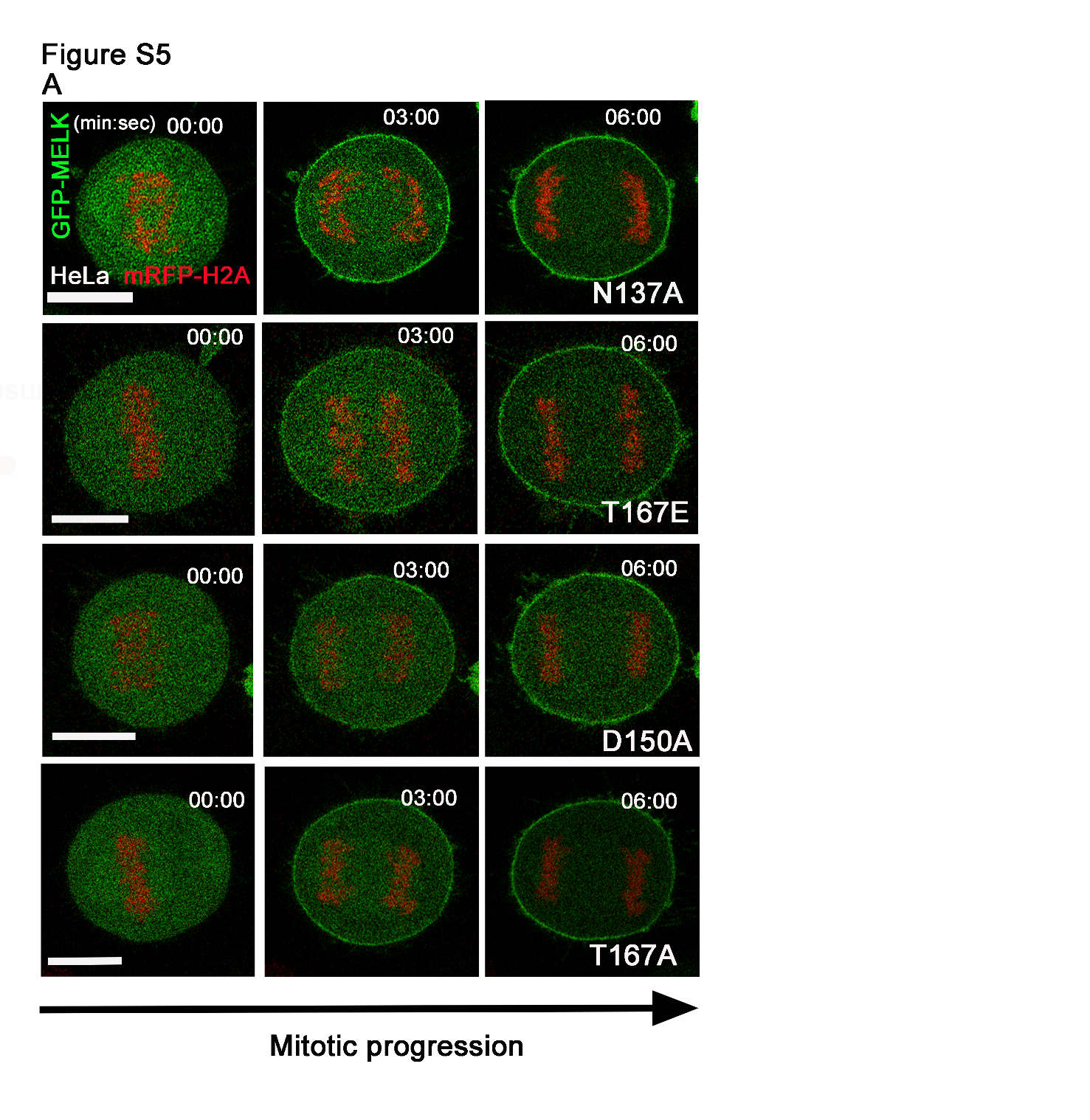


**Figure S5. Cortical localization of various mutants of MELK.**

HeLa cells that stably express mRFP-histone H2A were transfected with four GFP-MELK mutants N137A, T167E, D150A and T167A. Single plane images are shown with time intervals of 3 min. Scale bar is 10µm**.**

**
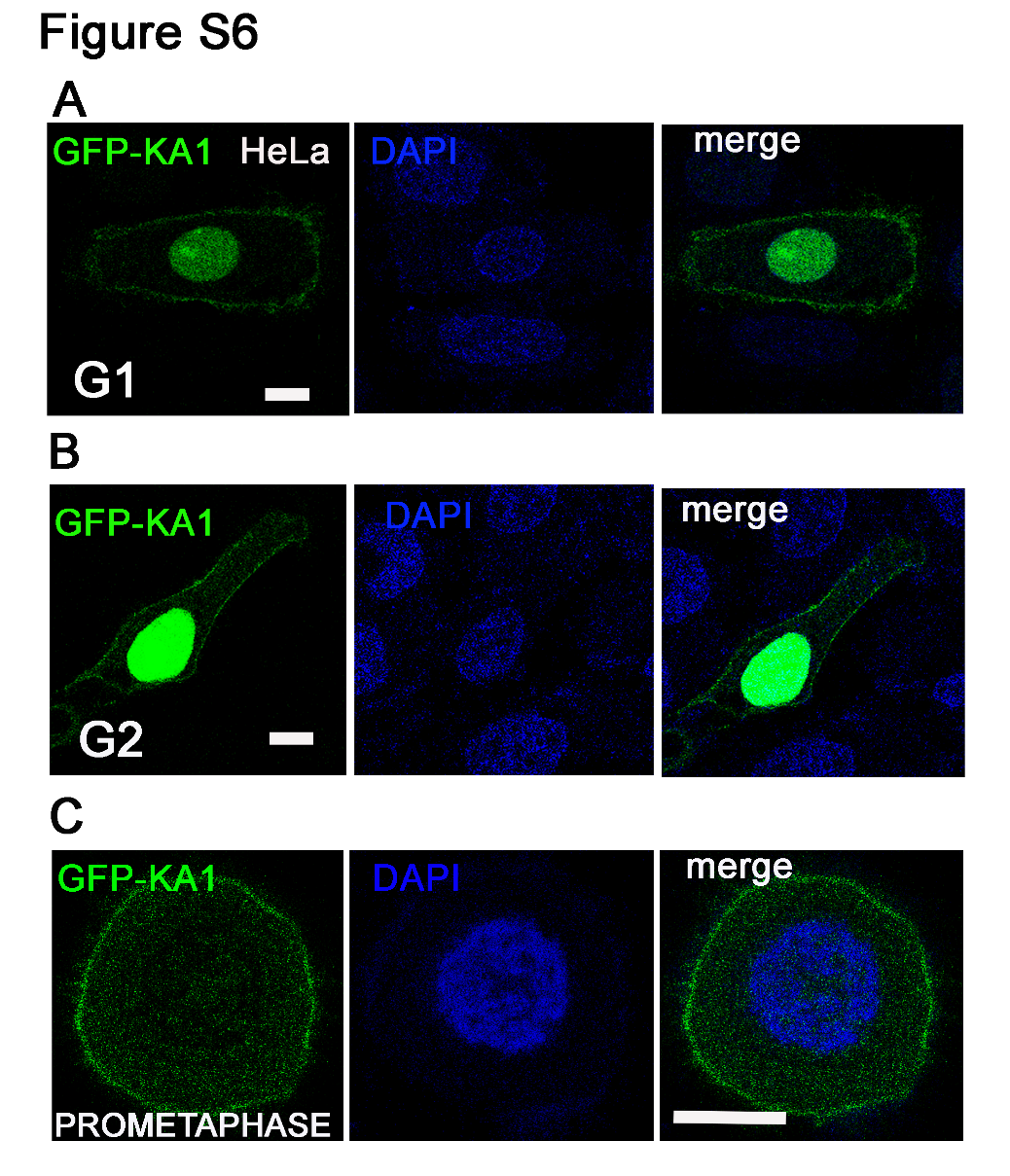
**

**Figure S6. MELK-KA1 domain is associated with the cell cortex throughout the cell cycle.**

HeLa cells transfected with GFP-KA1 were synchronized at G1/S (by thymidine) (A), G2 (B) and prometaphase (C) (attached and mitotic cells after thymidine release into nocodazole for 12h). KA1 localization is constitutive at all stages (G1/S, G2, Prometaphase). The cells were fixed and counter-stained with DAPI for DNA. Note the distribution of GFP-KA1 signals on the cell cortex and also in the nuclei in G1 and G2 cells. More than 20 cells were examined for each stage.

**

**

**Figure S7. GFP quantification for Fig 3 experiments.**

Related to Fig 3. GFP expression levels in HeLa cells transfected with wild type (WT) GFP-MELK, GFP-MELK-DM, GFP-MELKΔKA1, and GFP-KA1 were quantified using the maximum projections of metaphase cells, and shown in bar-scatter plot with mean±SD.

**
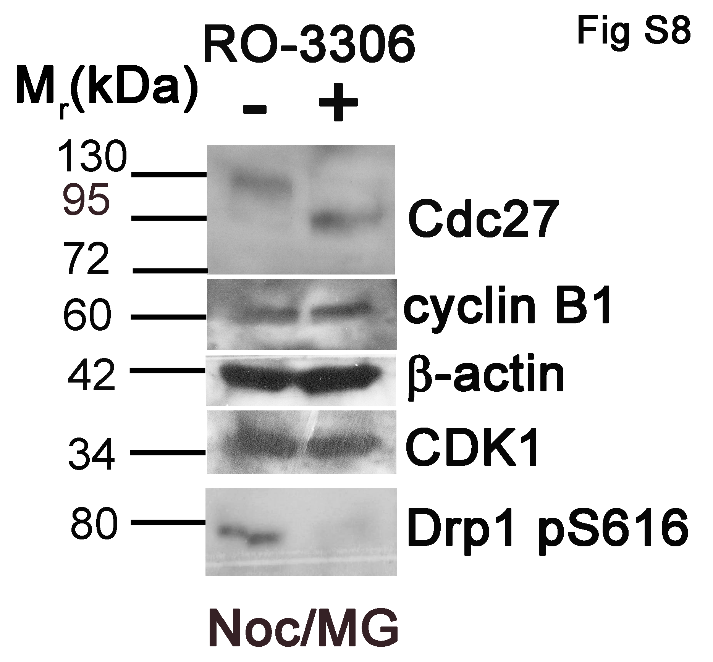
**

**Figure S8. Related to Fig 4.** **RO-3306 treatment for 1 hr of mitotic cells arrested in nocodazole/MG132 (Noc/MG) did not change CDK1 protein level.**

The lysates were prepared similarly as in Fig 4A after 1 hr RO-3306 treatment. β-actin was probed as the loading control. CDK1 and Cyclin B1 levels show no change in the presence of RO-3306. CDC27, a subunit of the Anaphase Promoting Complex/Cyclosome (APC/C), is a well-characterized CDK1 substrate whose phosphorylation at multiple sites results in well-known dramatic mobility shift on SDS-PAGE (Fujimitsu, Grimaldi et al. 2016, Zhang, Chang et al. 2016). RO-3306 treatment resulted in the loss of CDC27 mobility shift, indicating loss of CDK1 phosphorylation on CDC27. The blot was stripped and re-probed with anti-Drp1 Phospho-Ser616 antibody (Cell Signaling #3455). Drp1 Ser616 phosphorylation is mediated by CDK1 during mitosis, leading to mitochondrial fragmentation during mitosis (Taguchi, Ishihara et al. 2007). This phospho-antibody also showed loss of phosphorylation of Drp1 at Ser616 when CDK1 was inhibited by RO-3306.

**
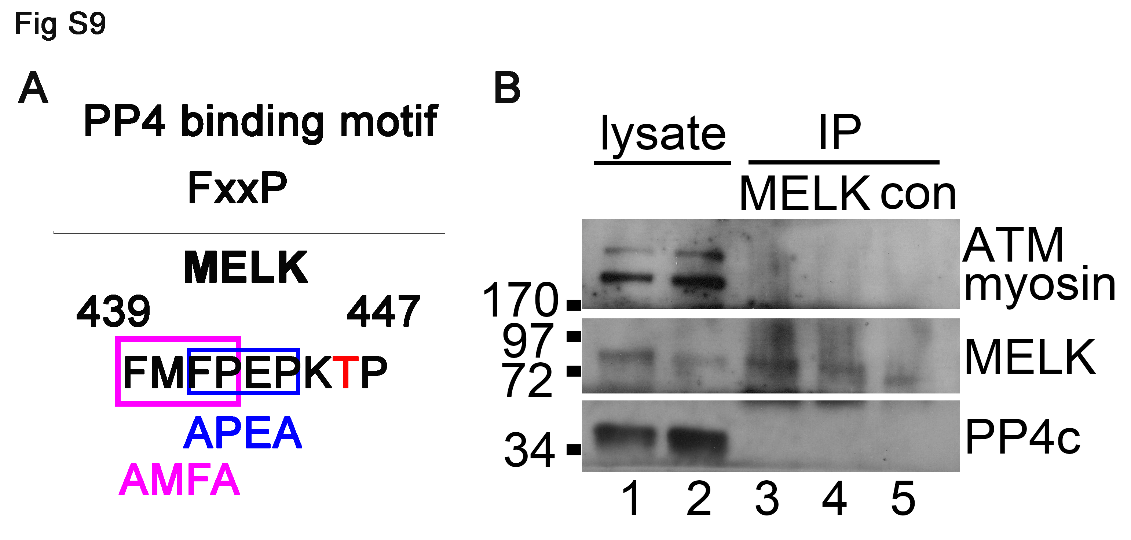
**

**Figure S9. Related to Fig 5.** **Examination of potential interactions between PP4 and MELK.**

1. Shown are two overlapping potential PP4 binding motifs within MELK TP region: FMFP and FPEP.
2. No PP4 catalytic subunit (PP4c) was observed in MELK immunoprecipates from prometaphase (nocodazole arrested, lane 3) and anaphase enriched (nocodazole released for 2 hours, lane 4) cell lysates. The same lysates were probed in lanes 1 and 2 respectively, while lane 5 was control IgG (con) immunoprecipitation from nocodazole lysate. ATM and myosin were probed as loading controls. Molecular weight markers (kDa) are marked on the left.

**
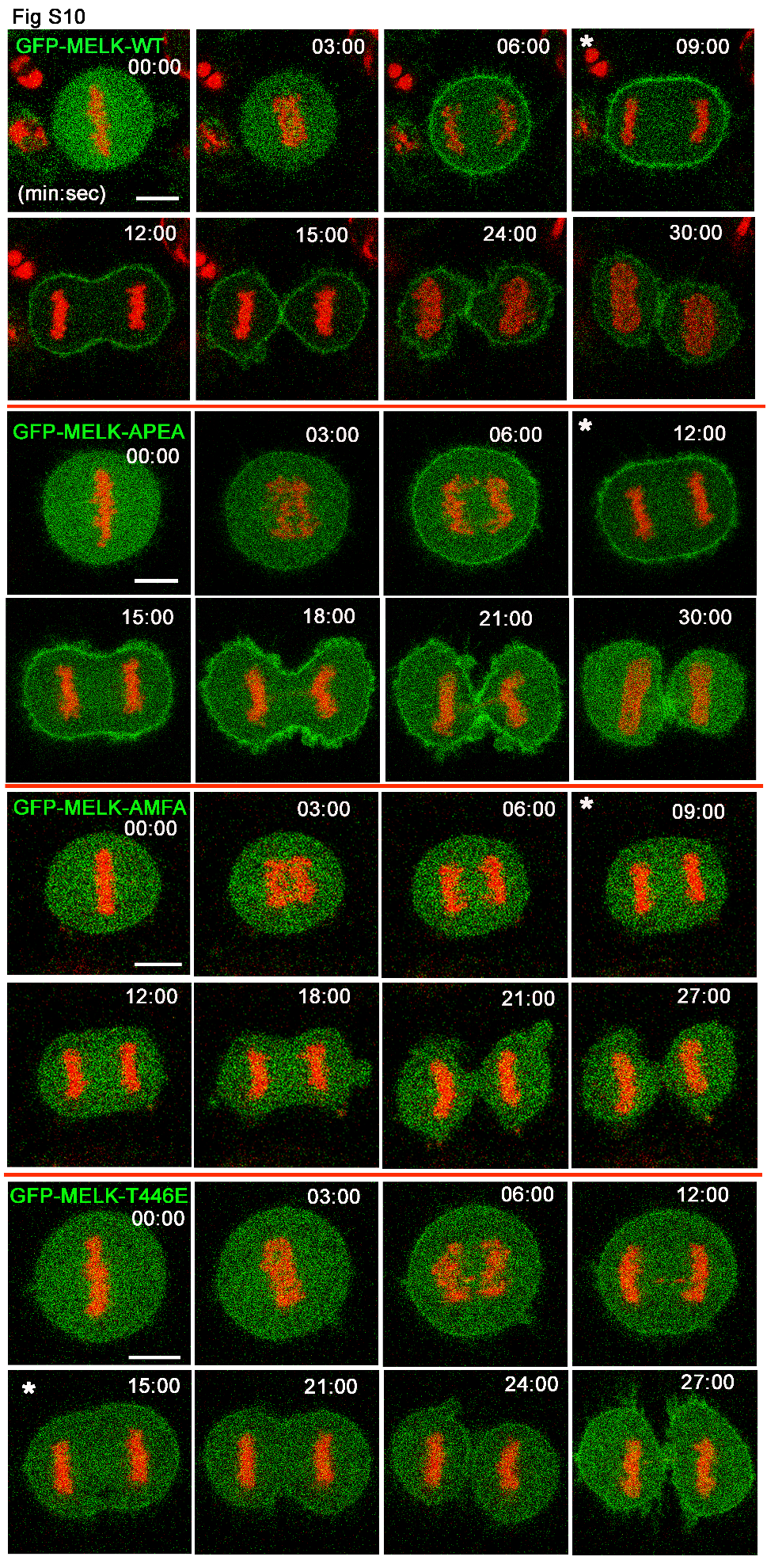
**

**Figure S10. Related to Fig 5.** **Selected images of MELK mutants at potential PP4 binding motifs or a regulatory site.**

Selected images from time lapse recording from metaphase to cytokinesis of HeLa cells transfected with GFP-MELK-wild type (WT), or its FPEP to APEA mutant, or FMFP to AMFA mutant, or T446E mutant. The cells also stably express mRFP-histone H2A. Time stamp, min: sec with last metaphase plate set as t=0. Scale bar = 10µm. The images marked with asterisks (*) were used in Fig 5A.

**Supplemental Tables**

Table S1. List of small molecule inhibitors

| **Small molecule inhibitors** | **Target kinases** | **Final concentration** | **Manufacturer catalog number** | **Reference** |
| --- | --- | --- | --- | --- |
| PD166326 | Src/Abl | 5µM | Sigma-Aldrich PZ0366 | (Wolff, Veach et al. 2005) |
| SB202190 | P38 MAPK | 10µM | Milipore Sigma S7067 | (Hirosawa, Nakahara et al. 2009) |
| MLN8237 | Aurora A kinase | 500nM | Cayman Chemicals 13602 | (Oku, Nishiya et al. 2018) |
| U0126 | MEK1/2 | 10µM | Promega V1121 | (Tao, Shi et al. 2019) |
| JNK-IN-8 | JNK1/2/3 | 5µM | Sigma-Aldrich  SML1246 | (Ebelt, Kaoud et al. 2017) |
| PD98059 | MAP kinase kinase (MEK) | 10µM | Sigma- Aldrich  513000 | (Zhao, Ge et al. 2017) |
| Hesperadin | Aurora B kinase | 100nM | Adooq bioscience A10448 | (Hauf, Cole et al. 2003) |
| PLK1 inhibitor III | Plk1 | 100nM | Calbiochem CAS 660868-91-7 | (Gleixner, Ferenc et al. 2010) |
| Roscovitine | CDK1 | 5µM | Calbiochem 557360 | (Wesierska-Gadek, Borza et al. 2009) |
| RO-3306 | CDK1 | 5µM | Sigma-Aldrich 217699 | (Vassilev, Tovar et al. 2006, Huang, Fan et al. 2023) |
| Reversine | MPS1 | 500nM | Calbiochem  R3904 | (Santaguida, Tighe et al. 2010) |
| MG132 | Proteasome inhibitor | 20µM | Cayman Chemicals 13697 | (Dang, Wen et al. 2014) |
| NVS-MELK8a | MELK inhibitor | 3µM | Med Chem Express  HY-100368 | (Toure, Giraldes et al. 2016) |
| HTH-01-091 | MELK inhibitor | 10µM | Gift from Dr. Nathanael Gray. | (Huang, Seo et al. 2017) |

Table S2. List of primers (mutant codons are underlined).

| **MELK mutants** | **Forward primer sequence 5’to 3’** | **Reverse primer sequence 5’to 3’** |
| --- | --- | --- |
| D150A | GAATATCATAAATTAAAGCTTGATTGCCTTTGGTCTCTGTGCAAAACCCAAG | CTTGGGTTTTGCACAGAGACCAAAGGCAATCAGCTTTAATTTATGATATTC |
| T167A | AGGATTACCATCTACAGGCATGCTGTGGGAGTCTG | CAGACTCCCACAGCATGCCTGTAGATGGTAATCCT |
| T167E | CTACAGGAATGCTGTGGGAGTCTGGCTTATGCAGCACCTGAGTTAATA | ACTCCCACAGCATTCCTGTAGATGGTAATCCTTGTTACCCTTGGGTTTTG |
| N137A | CACAGGGACCTCAAGCCAGAAGCTTTGCTGTTTGATGAATATC | GATATTCATCAAACAGCAAAGCTTCTGGCTTGAGGTCCCTGTG |
| RRLK TO SSSS | GACGGGCCCTCTTCTTCTTCTCTTCACTATAACGTGACTACAACTAGATTAGTGAAT | AGAAGAAGAAGAGGGCCCGTCTCTGGCAGAACCCTTCCTTTTGCTCCT |
| RRQR TO SSSS | GGTATCAGTAGTTCCTCGCTTAAGGGCGATGCCTGGGTTTACAAAAGATTAGTGGAA | CTTAAGCGAGGAACTACTGATACCCACCACATCGGGTTTTTGAAGCTGGCA |
| T478A | TGCCTGAAAGAAGCTCCAATTGCTCCAATTAAAATACCAG | CTGGTATTTTAATTGGAGCAATTGGAGCAATTGGAGCTTCTTTCAGGCA |
| T498A | ATGACAGGTGTCATTGCCCCTGAGAGGCGGTGCCGCTCA | TGAGCGGCACCGCCTCTCAGGGGCAATGACACCTGTTCAT |
| T460A, T466A | GAAATACTCACTGCGCCAAATCGTTACACTGCACCCTCAAAAGCTAGA | TCTAGCTTTTGAGGGTGCAGTGTAACGATTTGGCGCAGTGAGTATTTC |
| T517A | CAAGCACATATGGAGGAGGCTCCAAAAGAAGGAGCC | GGCTCCTTCTTTTGGAGCCTCCTCCATATGTGCTTG |
| T478E | AACCAGTGCCTGAAAGAAGAACCAATTAAAATACCAGTA | TACTGGTATTTTAATTGGTTCTTCTTTCAGGCACTGGTT |
| T517E | CAAGCACATATGGAGGAGGAACCAAAAAGAAAGGGAGCC | GGCTCCCTTTCTTTTTGGTTCCTCCTCCATATGTGCTTG |
| T460,T466E | GAAATACTCACGAACCAAATCGT TAC ACTGAACCCTCA AAAGCTAGA | TCTAGCTTTTGAGGGTTCAGTGTAACGATTTGGTTCAGTGAGTATTTC |
| T498E | ATGACAGGTGTCATTGAA CCT GAG AGGCGGTGCCGCTCA | TGAGCGGCACCGCCTCTCAGGTTCAATGACACCTGTCAT |
| FMFP TO AMFA | GTA AAG AAT GAA GAG TAC GCT ATG TTT GCTGAGCCAAGACTCCAG | CTGGAGTCTTGGCTCAGCAAACATAGCGTACTCTTCATTCTTTAC |
| FPEP TO APEA | GAATGAAGAGTACTTTATGGCTCCT  GAGGCAAAGACTCCAGTTAATAAG | CTTATTAACTGGAGTCTTTGCCTCAGGAGCCATAAAGTACTCTTCATTC |
| T446E | GTTTCCTGAGCCAAAGGAGCCAGTTAATAAGAAC | GTTCTTATTAACTGGCTCCTTTGGCTCAGGAAAC |

**Supplemental References**

Dang, L., F. Wen, Y. Yang, D. Liu, K. Wu, Y. Qi, X. Li, J. Zhao, D. Zhu, C. Zhang and S. Zhao (2014). "Proteasome inhibitor MG132 inhibits the proliferation and promotes the cisplatin-induced apoptosis of human esophageal squamous cell carcinoma cells." Int J Mol Med **33**(5): 1083-1088.

Ebelt, N. D., T. S. Kaoud, R. Edupuganti, S. Van Ravenstein, K. N. Dalby and C. L. Van Den Berg (2017). "A c-Jun N-terminal kinase inhibitor, JNK-IN-8, sensitizes triple negative breast cancer cells to lapatinib." Oncotarget **8**(62): 104894-104912.

Fujimitsu, K., M. Grimaldi and H. Yamano (2016). "Cyclin-dependent kinase 1-dependent activation of APC/C ubiquitin ligase." Science **352**(6289): 1121-1124.

Gleixner, K. V., V. Ferenc, B. Peter, A. Gruze, R. A. Meyer, E. Hadzijusufovic, S. Cerny-Reiterer, M. Mayerhofer, W. F. Pickl, C. Sillaber and P. Valent (2010). "Polo-like kinase 1 (Plk1) as a novel drug target in chronic myeloid leukemia: overriding imatinib resistance with the Plk1 inhibitor BI 2536." Cancer Res **70**(4): 1513-1523.

Hauf, S., R. W. Cole, S. LaTerra, C. Zimmer, G. Schnapp, R. Walter, A. Heckel, J. van Meel, C. L. Rieder and J. M. Peters (2003). "The small molecule Hesperadin reveals a role for Aurora B in correcting kinetochore-microtubule attachment and in maintaining the spindle assembly checkpoint." J Cell Biol **161**(2): 281-294.

Hirosawa, M., M. Nakahara, R. Otosaka, A. Imoto, T. Okazaki and S. Takahashi (2009). "The p38 pathway inhibitor SB202190 activates MEK/MAPK to stimulate the growth of leukemia cells." Leuk Res **33**(5): 693-699.

Huang, H. T., H. S. Seo, T. Zhang, Y. Wang, B. Jiang, Q. Li, D. L. Buckley, B. Nabet, J. M. Roberts, J. Paulk, S. Dastjerdi, G. E. Winter, H. McLauchlan, J. Moran, J. E. Bradner, M. J. Eck, S. Dhe-Paganon, J. J. Zhao and N. S. Gray (2017). "MELK is not necessary for the proliferation of basal-like breast cancer cells." Elife **6**.

Huang, Y., Y. Fan, Z. Zhao, X. Zhang, K. Tucker, A. Staley, H. Suo, W. Sun, X. Shen, B. Deng, S. R. Pierce, L. West, Y. Yin, M. J. Emanuele, C. Zhou and V. Bae-Jump (2023). "Inhibition of CDK1 by RO-3306 Exhibits Anti-Tumorigenic Effects in Ovarian Cancer Cells and a Transgenic Mouse Model of Ovarian Cancer." Int J Mol Sci **24**(15).

Oku, Y., N. Nishiya, S. Sugiyama, H. Sato and Y. Uehara (2018). "Sensitisation of Cancer Cells to MLN8237, an Aurora-A Inhibitor, by YAP/TAZ Inactivation." Anticancer Res **38**(6): 3471-3476.

Santaguida, S., A. Tighe, A. M. D'Alise, S. S. Taylor and A. Musacchio (2010). "Dissecting the role of MPS1 in chromosome biorientation and the spindle checkpoint through the small molecule inhibitor reversine." J Cell Biol **190**(1): 73-87.

Taguchi, N., N. Ishihara, A. Jofuku, T. Oka and K. Mihara (2007). "Mitotic phosphorylation of dynamin-related GTPase Drp1 participates in mitochondrial fission." J Biol Chem **282**(15): 11521-11529.

Tao, M., Y. Shi, L. Tang, Y. Wang, L. Fang, W. Jiang, T. Lin, A. Qiu, S. Zhuang and N. Liu (2019). "Blockade of ERK1/2 by U0126 alleviates uric acid-induced EMT and tubular cell injury in rats with hyperuricemic nephropathy." Am J Physiol Renal Physiol **316**(4): F660-F673.

Toure, B. B., J. Giraldes, T. Smith, E. R. Sprague, Y. Wang, S. Mathieu, Z. Chen, Y. Mishina, Y. Feng, Y. Yan-Neale, S. Shakya, D. Chen, M. Meyer, D. Puleo, J. T. Brazell, C. Straub, D. Sage, K. Wright, Y. Yuan, X. Chen, J. Duca, S. Kim, L. Tian, E. Martin, K. Hurov and W. Shao (2016). "Toward the Validation of Maternal Embryonic Leucine Zipper Kinase: Discovery, Optimization of Highly Potent and Selective Inhibitors, and Preliminary Biology Insight." J Med Chem **59**(10): 4711-4723.

Vassilev, L. T., C. Tovar, S. Chen, D. Knezevic, X. Zhao, H. Sun, D. C. Heimbrook and L. Chen (2006). "Selective small-molecule inhibitor reveals critical mitotic functions of human CDK1." Proc Natl Acad Sci U S A **103**(28): 10660-10665.

Wesierska-Gadek, J., A. Borza, O. Komina and M. Maurer (2009). "Impact of roscovitine, a selective CDK inhibitor, on cancer cells: bi-functionality increases its therapeutic potential." Acta Biochim Pol **56**(3): 495-501.

Wolff, N. C., D. R. Veach, W. P. Tong, W. G. Bornmann, B. Clarkson and R. L. Ilaria, Jr. (2005). "PD166326, a novel tyrosine kinase inhibitor, has greater antileukemic activity than imatinib mesylate in a murine model of chronic myeloid leukemia." Blood **105**(10): 3995-4003.

Zhang, S., L. Chang, C. Alfieri, Z. Zhang, J. Yang, S. Maslen, M. Skehel and D. Barford (2016). "Molecular mechanism of APC/C activation by mitotic phosphorylation." Nature **533**(7602): 260-264.

Zhao, Y., C. C. Ge, J. Wang, X. X. Wu, X. M. Li, W. Li, S. S. Wang, T. Liu, J. Z. Hou, H. Sun, D. Fang and S. Q. Xie (2017). "MEK inhibitor, PD98059, promotes breast cancer cell migration by inducing beta-catenin nuclear accumulation." Oncol Rep **38**(5): 3055-3063.
